# Induction of cell death in ovarian cancer cells by doxorubicin and oncolytic vaccinia virus is associated with CREB3L1 activation

**DOI:** 10.1016/j.omto.2021.04.014

**Published:** 2021-04-29

**Authors:** Anna Mistarz, Matthew Graczyk, Marta Winkler, Prashant K. Singh, Eduardo Cortes, Anthony Miliotto, Song Liu, Mark Long, Li Yan, Aimee Stablewski, Kieran O’Loughlin, Hans Minderman, Kunle Odunsi, Hanna Rokita, A.J. Robert McGray, Emese Zsiros, Danuta Kozbor

**Affiliations:** 1 Department of Immunology, Roswell Park Comprehensive Cancer Center, Buffalo, NY 14263, USA; 2 Center for Personalized Medicine, Roswell Park Comprehensive Cancer Center, Buffalo, NY 14263, USA; 3 Department of Biostatistics and Bioinformatics, Roswell Park Comprehensive Cancer Center, Buffalo, NY 14263, USA; 4 Department of Gynecologic Oncology, Roswell Park Comprehensive Cancer Center, Buffalo, NY 14263, USA; 5 Department of Molecular and Cellular Biology, Roswell Park Comprehensive Cancer Center, Buffalo, NY 14263, USA; 6 Department of Flow and Image Cytometry, Roswell Park Comprehensive Cancer Center, Buffalo, NY 14263, USA; 7 Faculty of Biochemistry, Biophysics, and Biotechnology, Jagiellonian University, Kraków, Poland

**Keywords:** doxorubicin, CREB3L1, oncolytic vaccinia virus, ovarian cancer, IFN**-**β

## Abstract

We have demonstrated that oncolytic vaccinia virus synergizes with doxorubicin (DOX) in inducing immunogenic cell death in platinum-resistant ovarian cancer cells and increases survival in syngeneic and xenograft tumor models. However, the mechanisms underlying the virus- and doxorubicin-mediated cancer cell death remain unknown. In this study, we investigated the effect of the oncolytic virus and doxorubicin used alone or in combination on activation of the cytoplasmic transcription factor CREB3L1 (cyclic AMP [cAMP] response element-binding protein 3-like 1) in ovarian cancer cell lines and clinical specimens. We demonstrated that doxorubicin-mediated cell death in ovarian cancer cell lines was associated with nuclear translocation of CREB3L1 and that the effect was augmented by infection with oncolytic vaccinia virus or treatment with recombinant interferon (IFN)-β used as a viral surrogate. This combination treatment was also effective in mediating nuclear translocation of CREB3L1 in cancer cells isolated from ovarian tumor biopsies at different stages of disease progression. The measurement of CREB3L1 expression in clinical specimens of ovarian cancer revealed lack of correlation with the stage of disease progression, suggesting that understanding the mechanisms of nuclear accumulation of CREB3L1 after doxorubicin treatment alone or in combination with oncolytic virotherapy may lead to the development of more effective treatment strategies against ovarian cancer.

## Graphical abstract

**Figure:**
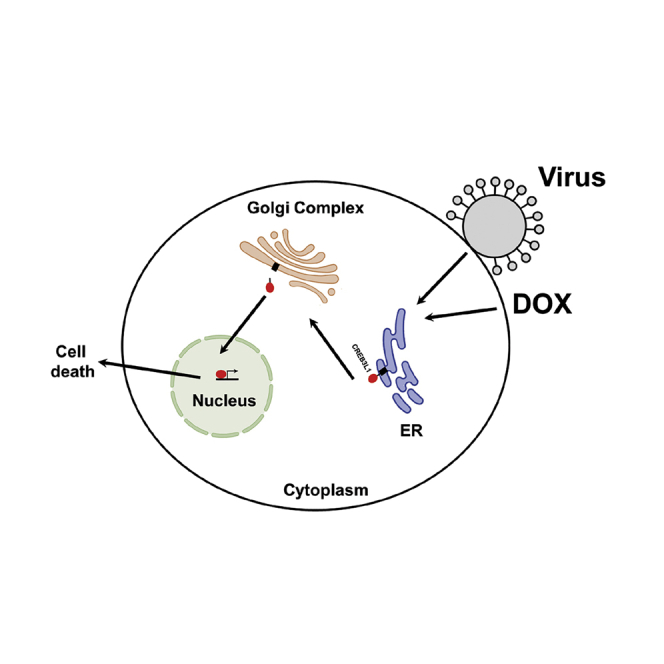

## Teaser

Induction of immunogenic cell death by doxorubicin in combination with oncolytic vaccinia virus is associated with activation of the cytoplasmic transcription factor CREB3L1.

## Introduction

Ovarian cancer (OC), often known as the silent killer, is the leading cause of death from gynecological malignancies.^1^ Most patients at the time of diagnosis present with metastatic disease, for which debulking surgery and chemotherapy are often not curative, resulting in recurrent disease within 5 years of diagnosis.^2^^,^^3^ Conventional second-line cytotoxic chemotherapies, including doxorubicin (DOX), achieve limited clinical benefit with overall response rates ranging from 10% to 25%,^4^ highlighting an unmet need for the development of novel therapies.^5^ Additionally, the tumor microenvironment (TME) of OC is highly immunosuppressive,^6^ suggesting that treatment strategies that engage the patients’ immune defense mechanisms through induction of immunogenic cell death (ICD) associated with type I interferon (IFN) production are important in contemporary cancer therapy. In this regard, oncolytic viruses (OVs) offer an attractive therapeutic combination of tumor-specific cell killing that is associated with immune stimulation.^7^ The approval of ICD-inducing pegylated liposomal DOX (Doxil) as a major component in the routine management of platinum-resistant epithelial OC (EOC)^8^ led to investigation of the combined treatment efficacy of oncolytic vaccinia virus (OVV) and DOX against platinum-resistant murine and human OC cells in syngeneic and xenograft OC models.^9^

Doxil is one of the best tolerated second-line treatments, which exerts not only a cytotoxic effect but can also enhance antitumor immune responses.^10^ DOX has been shown to inhibit proliferation of cancer cells through proteolytic activation of CREB3L1 (cyclic AMP [cAMP] response element-binding protein 3-like 1), a transcription factor synthesized as a membrane-bound precursor. CREB3L1 is a basic leucine zipper (bZIP) transcription factor of the CREB/ATF family with a transmembrane domain that allows it to associate with the endoplasmic reticulum (ER).^11^ Upon DOX treatment, CREB3L1 is cleaved by site-1 protease and site-2 protease, allowing the NH_2_-terminal domain of CREB3L1 to enter the nucleus where it activates transcription of genes encoding inhibitors of the cell cycle, including *p21.*^12^, ^13^, ^14^, ^15^ DOX was shown to inhibit growth of cells expressing *CREB3L1* but not those in which the gene was not expressed, even though the drug was equally effective in triggering DNA damage in both cells.^14^ Furthermore, knockdown of CREB3L1 mRNA in human hepatoma Huh7 cells conferred increased resistance to DOX, whereas overexpression of CREB3L1 in human breast cancer MCF-7 cells markedly enhanced DOX sensitivity in these cells.^14^ Epigenetic silencing of CREB3L1 by DNA methylation has been shown to be associated with high-grade metastatic breast cancers with poor prognosis and is prevalent in triple-negative breast^16^ and bladder^17^ carcinomas. A retrospective analysis performed on biopsy samples of triple-negative breast cancer indicated that CREB3L1 levels in tumors responsive to DOX chemotherapy were significantly higher than in the resistant counterparts.^15^ These results together with additional studies carried out in patients with advanced soft-tissue sarcoma suggested that measurement of CREB3L1 expression could be a useful biomarker in identifying cancer cells sensitive to DOX.^13^^,^^14^^,^^18^

The crucial role for CREB3L1 in inhibiting cell proliferation in response to DOX prompted us to examine whether activation of this transcription factor is involved during the induction of ICD by DOX and OVV in cancer cells. Oncolytic viruses, including OVV, mediate anticancer effects by direct oncolysis of cancer cells and stimulation of innate immune responses through production of damage-associated molecular patterns (DAMPs) and virus-derived pathogen-associated molecular patterns (PAMPs),^19^^,^^20^ leading to induction of type I IFN responses^21^ and improved T cell priming in EOC patients.^22^ Furthermore, an exogenous supply of type I IFNs was shown to restore chemotherapeutic responses to DOX in Toll-like receptor 3 (*Tlr3*)^−/−^ sarcomas growing in mice,^10^ supporting the use of IFN-β in augmenting DOX efficacy.^9^ In this study, we showed that DOX-mediated apoptosis of OC cell lines and clinical specimens was associated with translocation of CREB3L1 from the cytoplasm to nucleus and the effect was augmented by infection with OVV or treatment with IFN-β. Our findings also showed that CREB3L1 expression levels varied among OC patients and did not correlate with disease progression. These results suggest that the mechanisms of nuclear accumulation of CREB3L1 after DOX treatment alone or in combination with OVV might be explored for the development of more efficacious therapy against OC.

## Results

### Activity of the Western Reserve strain of oncolytic vaccinia in OC cells

**Figure 1:**
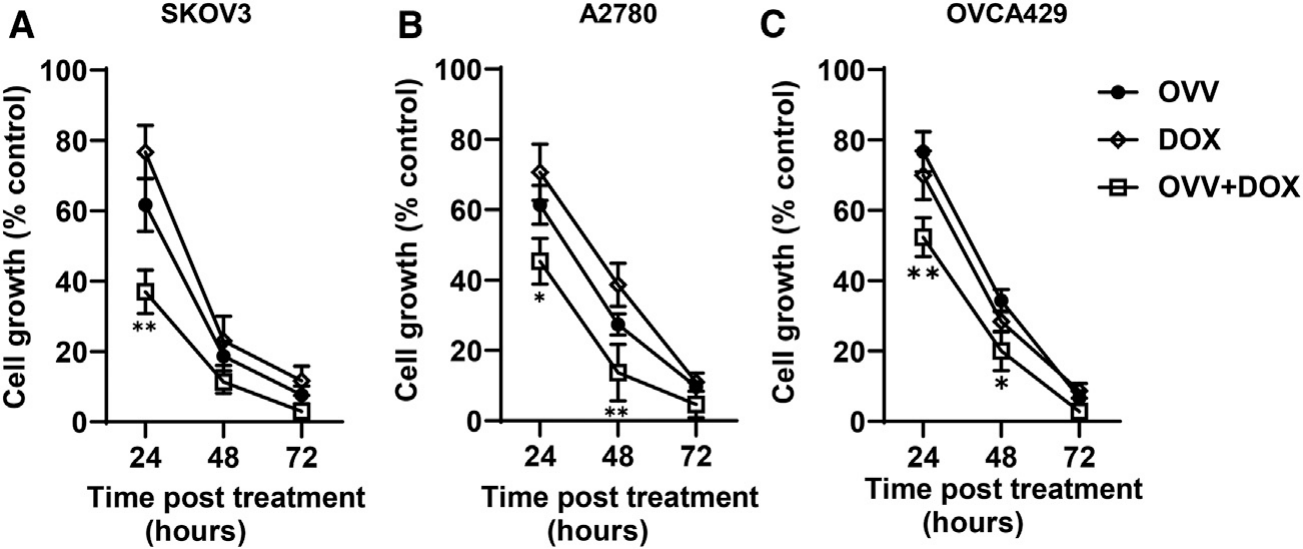
Inhibition of cell proliferation by DOX and OVV alone or in combination (A–C) On day 0, SKOV3 cells were seeded into 24-well culture plates at 1.9 × 10^5^ per well (A), A2780 cells were seeded at 3.8 × 10^5^ cells per well (B), and OVCA429 cells were seeded at 1.7 × 10^5^ cells per well (C). On day 1, the cultures were either infected with OVV (MOI of 1) for 2 h, incubated with DOX (1 μM), or treated with both consecutively and quantified to determine cell proliferation after 24, 48, and 72 h. For each cell line, the number of cells prior to the treatment and after treatment with no drug/virus was set to 0% and 100%, respectively. Results are presented as mean ± SD of three independent experiments. ∗p < 0.05, ∗∗p < 0.01.

**Figure 2:**
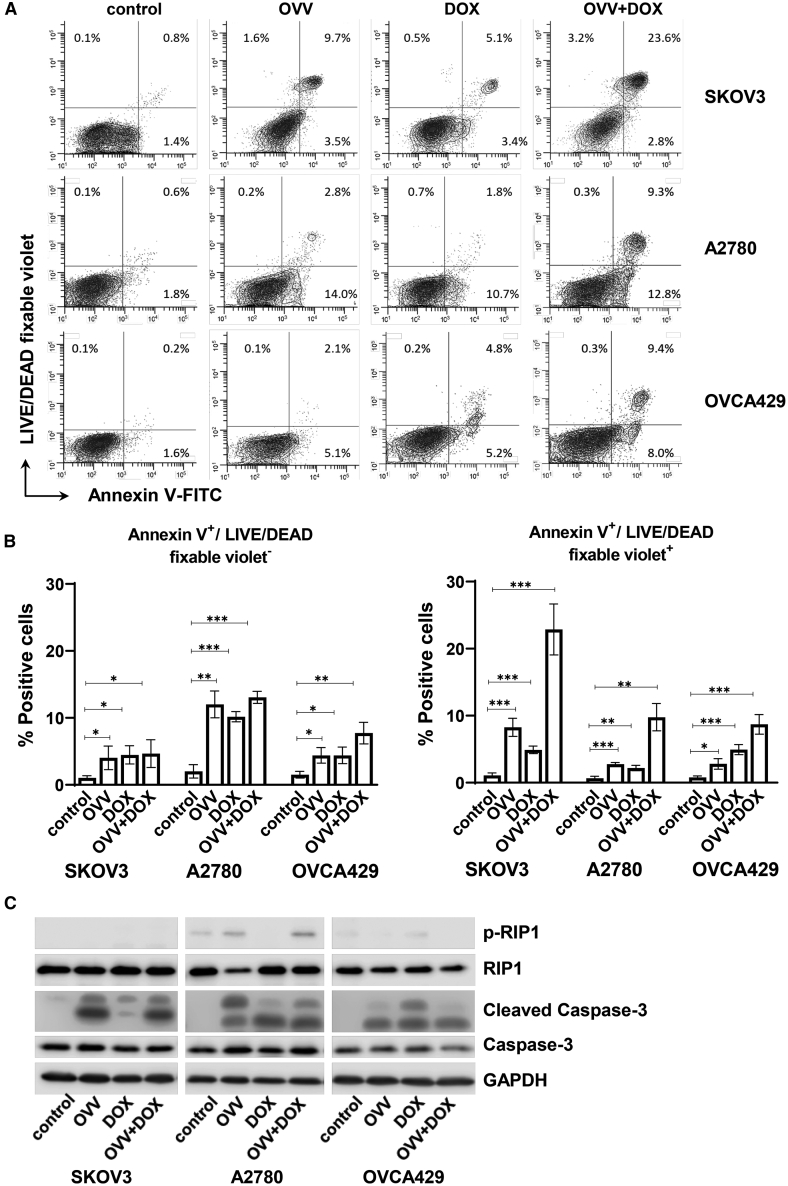
Effect of DOX and vaccinia virus on cell death in ovarian cancer cell lines (A) Cells were either infected with OVV (MOI of 1) for 2 h, incubated with 1 μM DOX for 16 h, or treated with both consecutively. Cultures were stained with Live/Dead fixable violet and incubated with annexin V-FITC. One representative experiment of three performed is shown. (B) (Left panel) Graph displaying the mean percentage of annexin V^+^/Live/Dead fixable violet^-^ cells. (Right panel) The mean percentage of annexin V^+^/Live/Dead fixable violet^+^ double-positive cells. ∗p < 0.05, ∗∗p < 0.01, ∗∗∗p < 0.001. (C) Western blot analysis of apoptotic and necroptotic markers in cell lysates harvested from untreated cells or cells treated with OVV, DOX, or OVV/DOX combination. Cell lysates were harvested and subjected to SDS-electrophoresis followed by immunoblotting with antibodies specific for receptor-interacting protein kinase 1 (RIP1), the phosphorylated form of RIP1, caspase-3, and cleaved caspase-3. GAPDH was used as a loading control. Representative blot of two experiments is shown.

We have previously reported that the ICD-inducing combination treatment consisting of the thymidine kinase (*TK*)- and vaccinia growth factor (*VGF*)-deleted strain of the Western Reserve (WR) virus and DOX resulted in synergistic killing of paclitaxel- and carboplatin-resistant murine and human OC cells *in vitro* and increased survival in tumor-bearing syngeneic mice that was associated with induction of antitumor immunity.^9^ Using an extended panel of human OC cell lines, including SKOV3, A2780, and OVCA429, we investigated the efficacy of the combined OVV and DOX treatment on inhibition of cell proliferation. As shown in Figures 1A–1C, treatment of OC cell lines with OVV at a multiplicity of infection (MOI) of 1 and DOX (1 μM) resulted in significantly higher inhibition of cell proliferation compared to each treatment used alone. The differences in sensitivity of the cancer cells to the combination treatments, which consisted of a sequential order of OVV added 2 h before DOX, were most prominent after 24 h (p < 0.05), whereas differences in cell growth between individual cultures gradually decreased 48 h after treatment and resulted in less than 10% viability by 72 h. To explore the cellular mechanisms involved in the increased inhibition of cell growth by the OVV and DOX treatment combination, we next investigated the induction of cancer cell death by OVV and DOX in SKOV3, A2780, and OVCA429 cultures. Cells were treated with OVV at an MOI of 1 for 2 h or DOX (1 μM) for 1 h alone or in combination. In the latter treatment, the virus was added 2 h before incubation with DOX for 1 h, and cells were analyzed 16 h later for the induction of cell death by flow cytometry with annexin V-fluorescein isothiocyanate (FITC) and Live/Dead fixable violet. As shown in Figures 2A and 2B, the numbers of annexin V^+^/Live/Dead fixable violet^-^ apoptotic cells^23^ increased in all cell cultures following OVV infection and DOX treatments alone or in combination (p < 0.05), whereas the numbers of annexin V^+^/Live/Dead fixable violet^+^ double-positive cells varied between individual cell lines with the highest percentages detected in SKOV3 cells. Alternatively, less than 5% of annexin^−^/Live/Dead fixable violet^+^ dead cells were detected in SKOV3 cells treated with the OVV and DOX combination, whereas their numbers were at background levels in both A2780 and OVCA429 cells.

Because classical apoptosis and necroptosis have been implicated in vaccinia virus infection to varying degrees,^24^ depending on the strain of the virus and molecular mechanisms that may differ in various cells,^25^ we next analyzed expression of cleaved caspase-3 as well as phosphorylated receptor-interacting protein kinase (RIP1) in cell lysates prepared from the OVV- and/or DOX-treated cancer cells, which would help to differentiate between the induction of apoptosis and necroptosis, respectively.^26^ Consistent with the increased frequency of annexin V^+^ cells in the treated cultures, cleavage of caspase-3 that is generally considered as a universal marker of apoptosis^27^ was apparent in all treated cells, whereas minimal phosphorylation of RIP1 was detected only in untreated and treated A2780 cancer cells (Figure 2C). Thus, our results support the previous reports of the induction of apoptosis in cancer cells by the WR strain-specific vaccinia,^25^^,^^28^^,^^29^ which differs from necroptotic cell death induced by Lister strain *TK*-deleted VV and a chimeric orthopoxvirus infection in ovarian and colorectal cancer cells.^24^^,^^30^

### Increased nuclear translocation of CREB3L1 by a combination treatment of OVV and DOX

**Figure 3:**
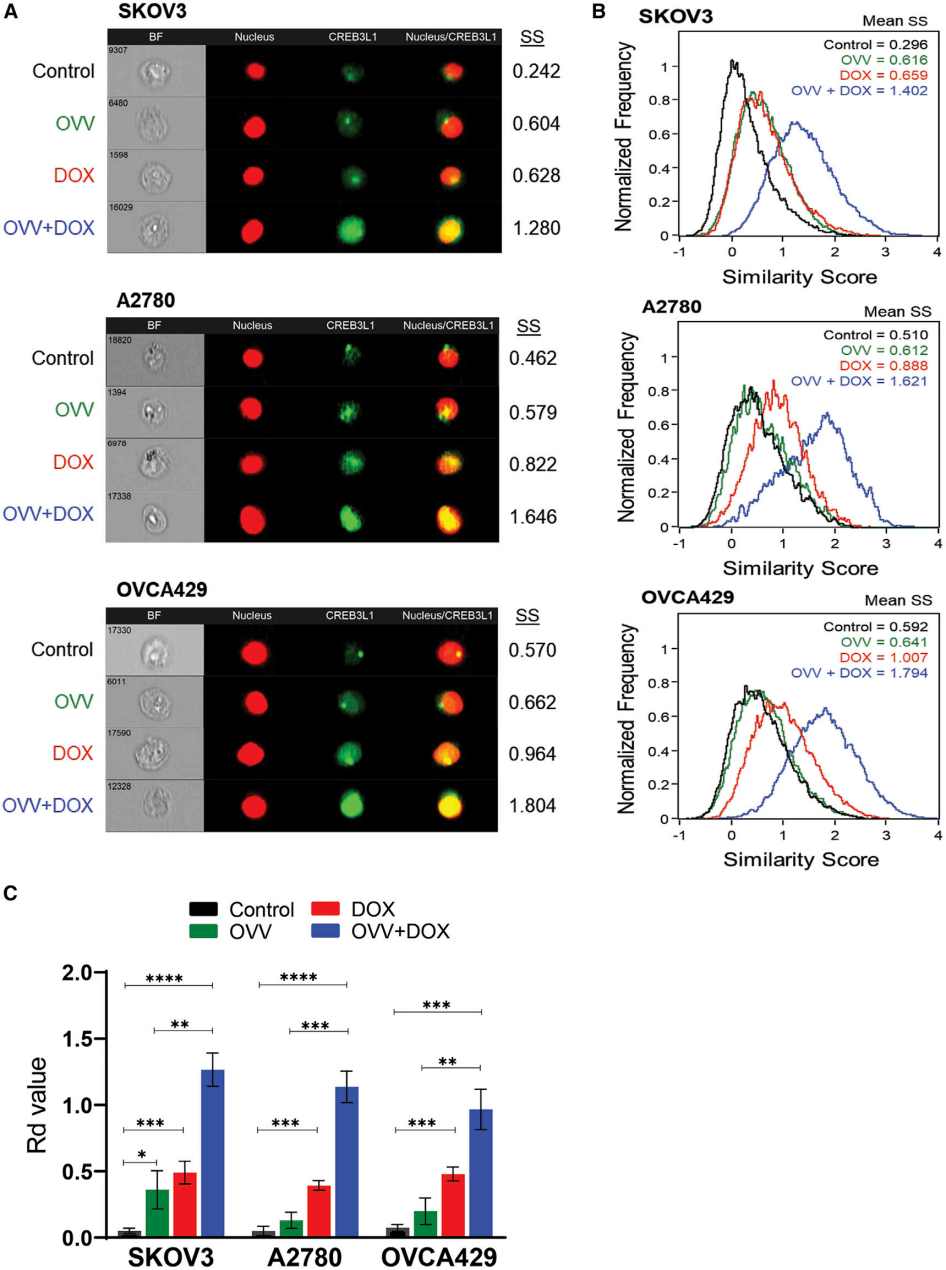
Effect of DOX and OVV on a nuclear localization of CREB3L1 in ovarian cancer cell lines by ImageStream analysis (A) SKOV3, A2780, and OVCA429 cells were either infected with OVV at an MOI of 1 for 2 h, incubated with DOX (1 μM, for 1 h), or treated with both consecutively and the extent of CREB3L1 translocation to DRAQ5-stained nuclei was monitored by staining with FITC-conjugated CREB3L1-specific antibody. Untreated and treated cells were visualized by ImageStream, and representative cells are shown (BF denotes brightfield image, DRAQ5 denotes nucleus, FITC denotes CREB3L1). (B) Distributions of cells with varying SSs are graphed. Translocation of CREB3L1 into the nucleus, measured by SS, significantly increased when cells were treated with OVV and DOX. (C) The Rd values are shown for each population. Results are reported as mean ± SD of three independent experiments. ∗p < 0.05, ∗∗p < 0.01, ∗∗∗p < 0.001, ∗∗∗∗p < 0.0001.

We next examined whether the induction of cell death in the OC cells by OVV and DOX alone or in combination was associated with a nuclear translocation of CREB3L1 from cytoplasm to the nucleus using ImageStream analysis. SKOV3, A2780, and OVCA429 cells were either infected with OVV at an MOI of 1 for 2 h, incubated with DOX (1 μM, for 1 h), or treated with both consecutively, and the CREB3L1 translocation to DRAQ5-stained nuclei was monitored by staining with FITC-conjugated CREB3L1-specific antibody, as visualized in Figure 3A. Next, samples acquired with the ImageStream were assessed for the extent of nuclear translocation of CREB3L1 by determining the mean similarity scores (SSs) to compare cytoplasmic versus nuclear localization of CREB3L1 within a treatment group. The SS provides an assessment of the degree of nuclear localization of CREB3L1 by measuring on a single-cell basis the pixel intensity correlation between the anti-CREB3L1-FITC and corresponding DRAQ5 nuclear image. In general, cells with low SSs exhibit poor correlation between the images (corresponding with a predominant cytoplasmic distribution of CREB3L1), whereas cells with high SS scores exhibit a high correlation between the images (corresponding with a predominant nuclear distribution of CREB3L1). Thus, analyses of CREB3L1 expression in SKOV3, A2780, and OVCA429 cells revealed the mean SS values for the control populations to range from 0.296 to 0.592, reflecting the relatively low similarity of FITC (CREB3L1) and DRAQ5 (nucleus) images, and therefore the predominant cytoplasmic localization of CREB3L1 (Figure 3B). The mean SSs for the treated populations varied, with the highest values achieved after the combined treatments, indicating a high similarity between the FITC and DRAQ5 images and increased localization of CREB3L1 to the nucleus.

The relative shift in SS distribution of CREB3L1 between two cell populations (e.g., control versus treated cells) was next quantified using the Fisher’s discriminant ratio (Rd) value as previously described.^31^ Figure 3C demonstrated only a small shift of CREB3L1 nuclear translocation in SKOV3 cells after OVV treatment (Figure 3C; p = 0.02). In both A2780 and OVCA429 cells, the responses were at background levels, suggesting that the cellular events occurring within the first 2 h of infection in these cells did not trigger the signaling cascade associated with accumulation of the transcription factor in the nucleus. Time-course analysis of CREB3L1 translocation at 1, 3, and 5 h after OVV treatment indicated lack of translocation (Figure S1), which was further confirmed when the time of analysis was extended to 24 h (data not shown). Alternatively, DOX treatment mediated a distinct increase in Rd values for the CREB3L1/nuclear SS distributions compared to control cells in all tested cell lines (Figure 3C; p < 0.05). The highest responses were measured after a combined treatment of OVV and DOX (p < 0.0001), which were significant compared to cultures treated with OVV or DOX alone (p < 0.01). The CREB3L1 translocation events monitored with ImageStream were further confirmed by western blotting of nuclear fractions prepared from cell lysates of SKOV3, A2780, and OVCA429 cells that were either untreated or treated with OVV and DOX alone or in combination using the same treatment conditions as for the ImageStream analysis. Immunoblotting of proliferating cell nuclear antigen (PCNA) was used as a loading control for nuclear extract and density of the CREB3L1 bands. As shown in Figure S2, the analysis confirmed increases in the nuclear localization of CREB3L1 after the combined OVV and DOX treatment, which was consistent with the previously reported correlation ImageStream assessment of receptor-mediated (tumor necrosis factor [TNF]-α) and drug (daunorubicin)-induced nuclear factor κB (NF-κB) translocation in leukemia cells with western blot analysis.^31^

**Figure 4:**
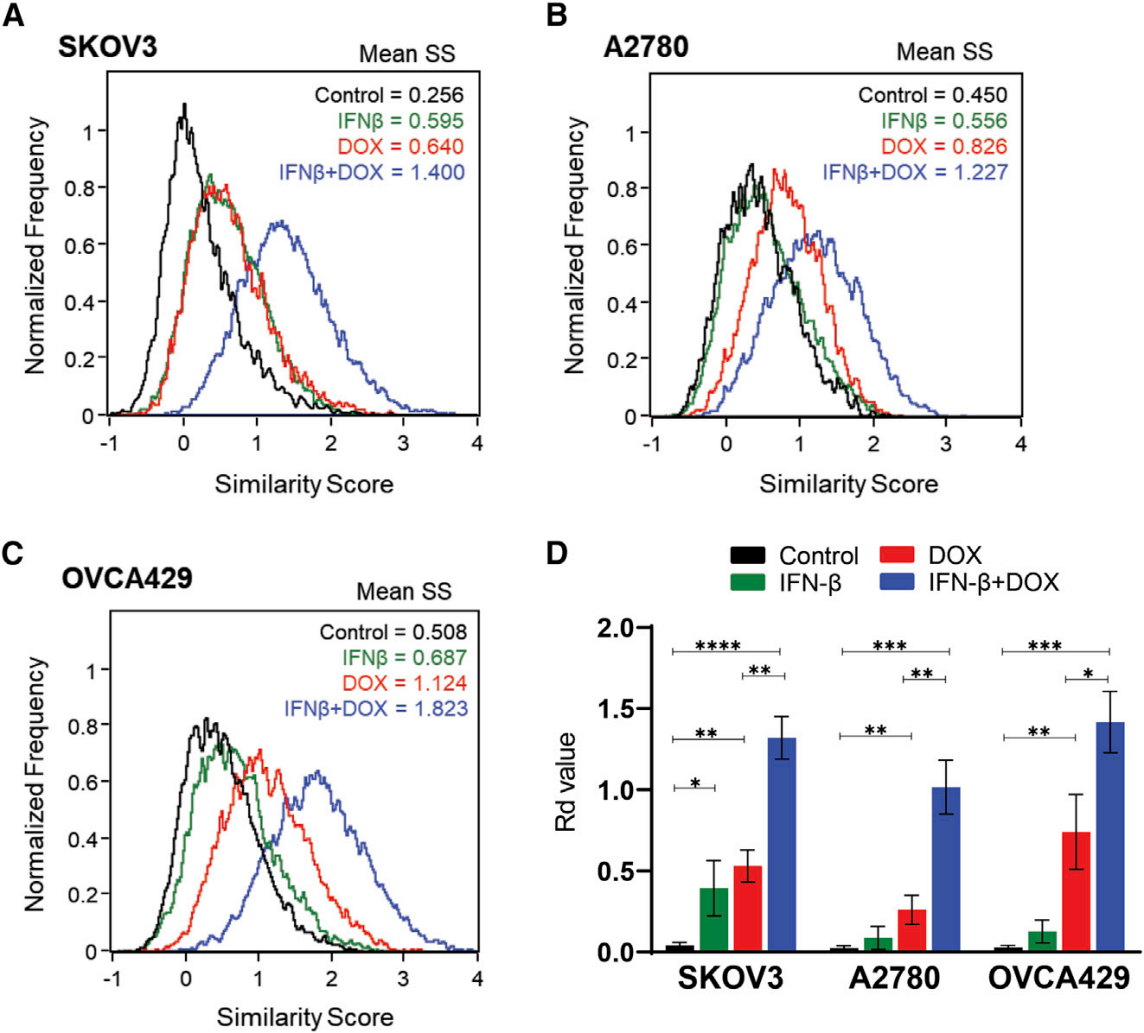
IFN-β-enhanced DOX-mediated CREB3L1 nuclear translocation in ovarian cancer cell lines (A–C) SKOV3 (A), A2780 (B), and OVCA429 (C) cell lines were treated with IFN-β (100 pg/mL) alone or in combination with 1 μM DOX and analyzed for translocation of CREB3L1 into the nucleus by ImageStream. Distributions of cells with varying SSs are graphed. (D) The Rd values are shown for each population. Results are presented as mean ± SD of three independent experiments. ∗p < 0.05, ∗∗p < 0.01, ∗∗∗p < 0.001, ∗∗∗∗p < 0.0001.

The previous findings that an exogenous supply of type 1 IFNs restored the chemotherapeutic responses to DOX in *Tlr3*^−/−^ but not IFN (α and β) receptor 2-deficient (*Ifnar2*^*−/−*^) sarcomas growing in mice,^10^ together with the ability of OVV-induced IFN-β in murine and human tumor cell cultures to augment responses to DOX,^9^ raised the possibility that IFN-β produced in response to OVV could enhance DOX-induced apoptosis mediated by CREB3L1 translocation to the nucleus. The levels of IFN-β measured by an enzyme-linked immunosorbent assay (ELISA) in culture supernatants harvested from SKOV3, A2780, and OVCA429 cells 48 h after infection with OVV revealed that the IFN-β levels varied from 30 to 90 pg/mL among the treated cell lines with the lowest and highest levels measured in OVCA429 and SKOV3 cells, respectively (Figure S3). To address the possibility that IFN-β could enhance DOX-induced apoptosis mediated by CREB3L1 translocation to the nucleus, the cancer cells were incubated with 100 pg/mL recombinant IFN-β alone or in combination with DOX and analyzed by ImageStream. As shown in Figures 4A–4C, the treatment with IFN-β had a small effect on CREB3L1 activation. However, it significantly augmented CREB3L1 nuclear translocation when used in combination with DOX compared to DOX alone (Figure 4D; p < 0.02), supporting the notion that optimal clinical responses to anthracycline-based chemotherapy rely on type I IFN signaling in neoplastic cells.^10^

### Analysis of CREB3L1 mRNA expression in human EOC clinical specimens

**Figure 5:**
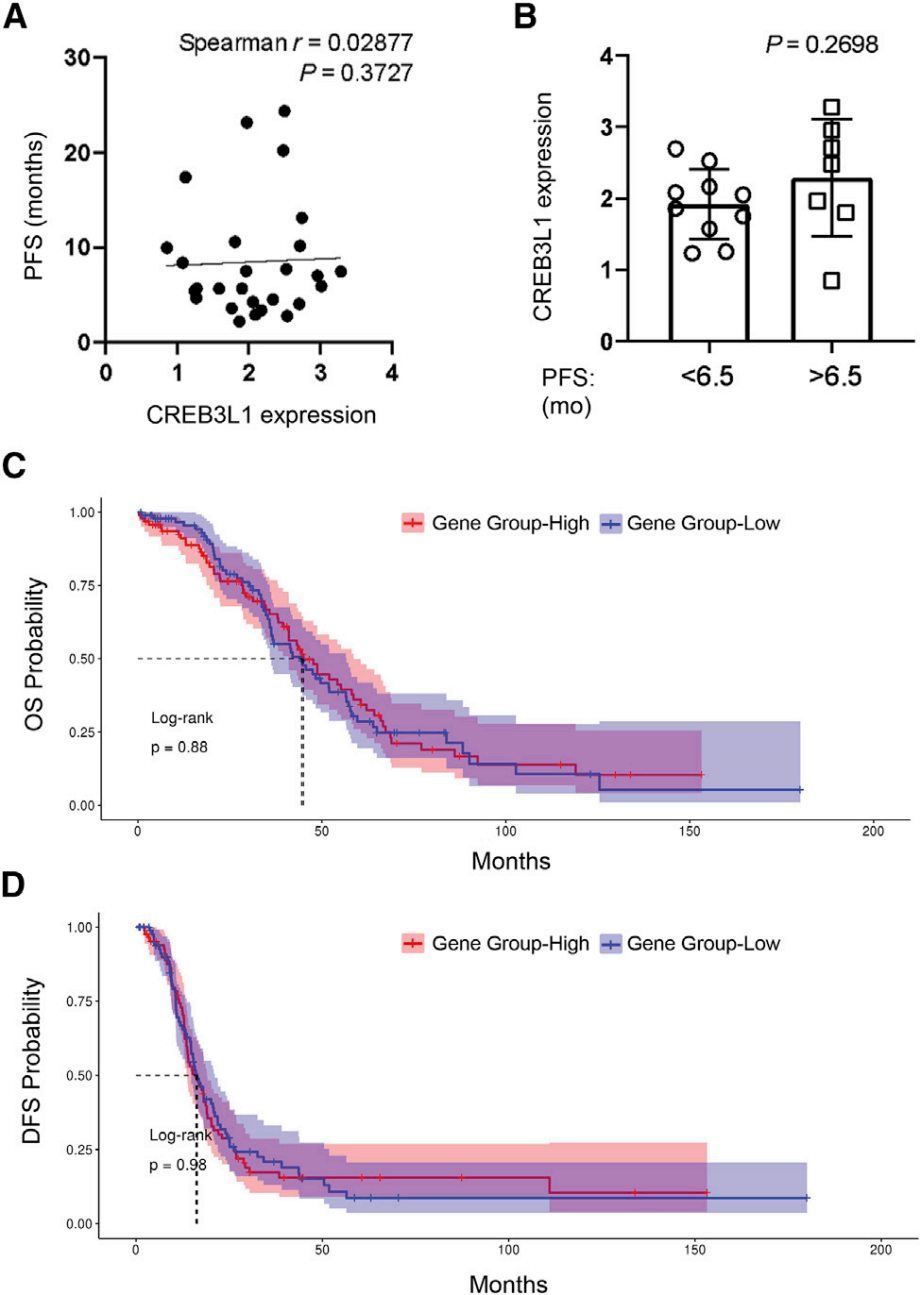
RNA-seq analyses of CREB3L1 expression in ovarian tumor data (A) Spearman correlation between PFS and CREB3L1 expression levels in biopsy samples taken from cohorts of 28 EOC patients with advanced stage of diseases with 17 of these patients treated with Doxil prior to entering clinical trials with anti-PD1 antibody. (B) Comparison of CREB3L1 expression levels splitting PFS by the median value of samples of patients treated with Doxil. (C and D) Kaplan-Meier analysis of OS (C) and DFS (D) in the cohort of EOC patients from TCGA database. CREB3L1 overexpression (red line) was not associated with higher OS (p = 0.88) and DFS (p = 0.98).

To investigate the heterogeneity of CREB3L1 expression in clinical specimens, we performed a retrospective RNA sequencing (RNA-seq) analysis of CREB3L1 expression levels in biopsy samples taken from cohorts of 28 OC patients with advanced stages of disease (23 out of 28 patients were resistant to carboplatin), with 17 of these patients treated with Doxil prior to entering a clinical trial for treatment with anti-PD1 antibody (ClinicalTrials.gov: NCT02853318). The CREB3L1 expression level was compared with progression-free survival (PFS) and the score of tumor-infiltrating lymphocytes (TILs), previously reported to be associated with improved PFS.^26^^,^^27^ The TIL scores, ranging from 1 to 3, were determined based on numbers of T cells per high-power field (HPF) with a score of 1 denoting 1–10 TILs per HPF, a score of 2 denoting 11–20 TILs per HPF, and a score of 3 denoting >20 TILs per HPF on average. Figure 5A shows that the CREB3L1 expression levels differed among patients. However, although half of these specimens showed expression of CREB3L1 higher than the mean value of 2.04 ± 0.65, there was not a significant correlation with PFS (Spearman p = 0.3727). Similarly, expression of CREB3L1 was not associated with the TIL score, as the values were similar in patients with low and intermediate numbers of TILs (TILs 1 and 2) and those of group 3 (high TILs) (Figure S4A). Similarly, an additional analysis of CREB3L1 levels in the 17 patients treated with Doxil prior to the anti-PD1 antibody therapy also revealed no significant differences in CREB3L1 between patients with PFS time shorter and longer than the median value of 6.5 months (Figure 5B). These findings indicated that CREB3L1 expression levels in the tumor specimens predict neither the stage of disease, tumor progression, or responsiveness to Doxil.

We also analyzed mRNA expression levels of CREB3L1 in a series of 300 patients with EOC from The Cancer Genome Atlas (TCGA) and correlated with overall survival (OS) and disease-free survival (DFS) status, respectively. As shown in Figures 5C and 5D, we observed no tendency toward association of CREB3L1 expression with either OS (p = 0.88) or DFS (p = 0.98). The correlation analysis with the PFS showed that overexpression of CREB3L1 also did not associate with PFS in patients with OC (Figure S4B; p = 0.81).

### Nuclear translocation of CREB3L1 in EOC specimens after OVV and DOX treatments

**Figure 6:**
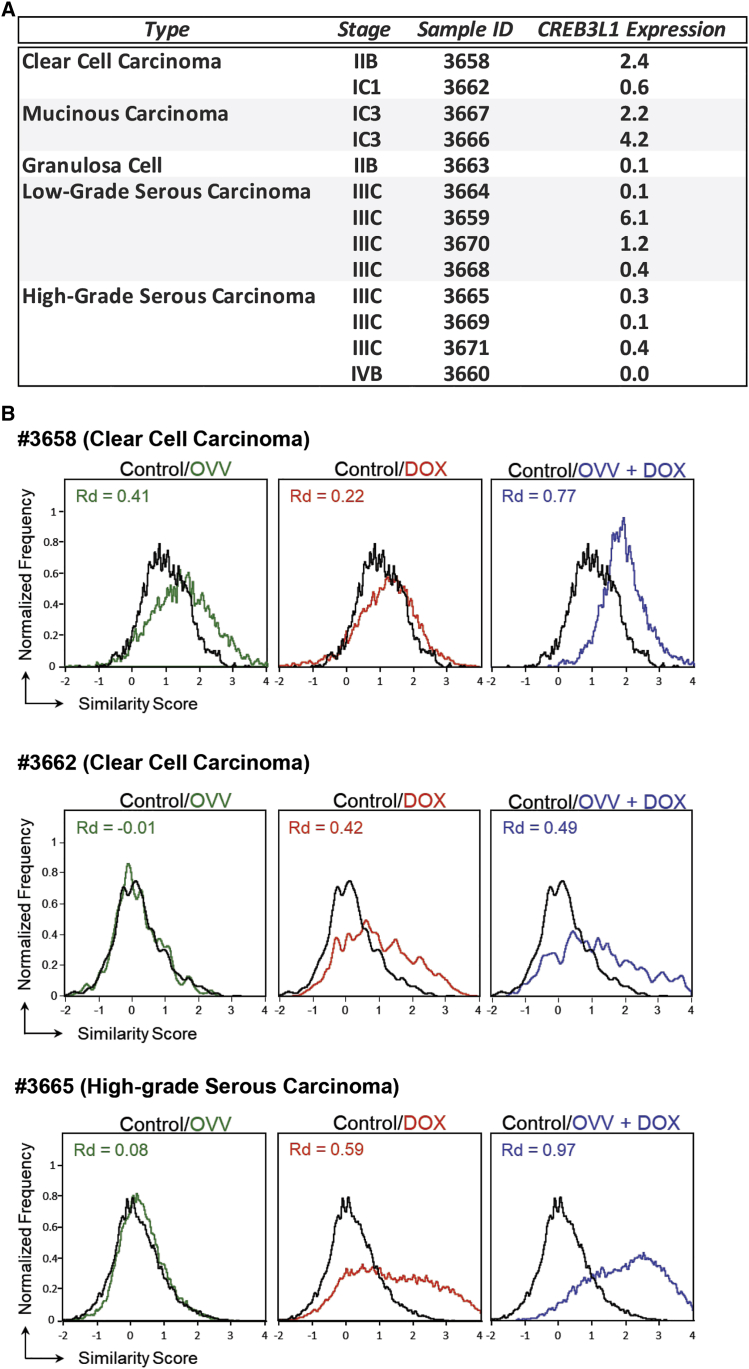
Analyses of CREB3L1 expression and activation in human ovarian tumor samples (A) The relative CREB3L1 expression in tumor samples representing different stages of EOC was determined by quantitative RT-PCR. (B) Differences in nuclear translocation of CREB3L1 in CD45-negative tumor cells isolated from representative clinical specimens after treatment with DOX and OVV alone or in combination were determined by the ImageStream analysis. The relative shifts in SS distributions of CREB3L1 between control and treated samples are graphed. The Rd values are shown for each population.

The heterogeneous expression levels of CREB3L1 in OC patients prompted us to examine both baseline expression and nuclear translocation of CREB3L1 in CD45-negative cancer cells after treatment with DOX, OVV, and a combination of OVV followed by DOX using clinical biopsies. The tumor tissues were isolated from patients with ovarian clear cell carcinoma, mucinous carcinoma, granulosa cells, as well as low- and high-grade serous carcinoma (International Federation of Gynecology and Obstetrics [FIGO] stage IC–IV) and analyzed for differences in CREB3L1 expression by quantitative real-time RT-PCR. The relative expression of CREB3L1, calculated using glyceraldehyde-3-phosphate dehydrogenase (GAPDH) as a reference gene, revealed a considerable variation among the analyzed specimens (Figure 6A). Only in 4 out of 13 analyzed specimens, including one patient with the stage IIIC low-grade serous carcinoma, were the relative expression levels of CREB3L1 higher than the mean value of 1.4 ± 1.9 SD (standard deviation), supporting the lack of association between OC disease subtype and disease stage.

To examine whether differences in CREB3L1 expression were associated with sensitivity of ovarian tumor cells to OVV and DOX treatment, we analyzed the treatment-induced nuclear translocation of CREB3L1 in specimens with different stages of tumor progression. For the study, CD45-negative cancer cells isolated from tumor biopsies of three patients (two with clear cell carcinoma [specimens #3662 and #3658] and one with high-grade serous carcinoma [specimen #3665]) were treated with OVV at an MOI of 1 (2 h) or DOX (1 μM for 1 h) alone in combination and analyzed by the end of the incubation period for nuclear translocation of CREB3L1 by ImageStream (Figure 6B). The extent of CREB3L1 activation after single and combined treatments relative to untreated controls was determined by Rd values and revealed a low-level response to OVV monotherapy only in one patient (#3658; Rd = 0.41 versus Rd < 0.1 for patients #3662 and #3665), whereas activation of CREB3L1 in DOX- or OVV/DOX-treated cells was observed, to varying degrees, in all patients. In patient #3662, the responses to DOX alone or used in combination with OVV were similar and higher than in the virally infected cells, whereas patient #3658 exhibited low responses to the virus or DOX alone but enhanced activation after the OVV/DOX combination. The most prominent nuclear translocation of CREB3L1 was demonstrated in cancer cells derived from patient #3665 (stage IIIC high-grade serous carcinoma), after treatment with DOX and the combination of OVV/DOX (Rd = 0.59 and 0.97, respectively), despite the lowest level of CREB3L1 expression among the analyzed specimens (Figure 6A). Altogether, the ImageStream results and the lack of correlation between the levels of CREB3L1 expression and PFS/DFS in clinical specimens suggest that the ability of patients with OC to respond to treatment with DOX does not depend on the CREB3L1 expression levels.

## Discussion

The current study establishes that inhibition of cellular proliferation of multiple human OC cell lines to treatment with DOX is augmented by infection with the WR strain of OVV and is associated with nuclear translocation of the CREB3L1 transcriptional factor. These results also support findings that the WR strain of vaccinia infection leads to apoptotic cell death,^25^^,^^28^^,^^29^ although (based on previous studies) this may be strain specific, as there was no apoptosis observed following Lister strain *TK-*deleted vaccinia and a chimeric orthopoxvirus infection in ovarian and colorectal cancer cells.^24^^,^^30^ While vaccinia virus encodes numerous inhibitors of apoptosis and ICD, including FIL, NIL, and SP1,^32^^,^^33^ that prevent premature cell death, these are not shared by all strains; SPI-1, for example, is expressed in WR, Lister, and Copenhagen strains, whereas SPI-2 is expressed in the WR strain only.^34^ Furthermore, there are widespread differences in the expression of proteins involved in cell death between normal and malignant cells as well as between different populations of cancer cells, which can affect the mechanism and efficacy of vaccinia virus-induced cell death. However, despite the differences in the mechanisms of the induction of ICD by different strains of the virus, many strains of OVV have undergone testing in multiple clinical trials,^25^ which offer some perspective regarding the development of next-generation OVV vectors for use as cancer therapeutics in combinatorial approaches. Such combination treatments will require coordinated strategies to synergistically augment tumor cell killing with simultaneous induction of ICD, reduction of intratumoral recruitment of immunosuppressive elements in favor of immunostimulatory signals (e.g., IL-12), and augmenting local tumor-specific T cell accumulation to overcome a non-T cell-inflamed TME to induce potent and durable antitumor immune responses. As pharmaceutical drugs, including DOX, can be combined with oncolytic virotherapy for improved therapeutic efficacy,^9^ it is expected that combinations of OVV and DOX will soon be investigated in a clinical setting.

We have recently reported that paclitaxel- and carboplatin-resistant human and murine OC cells were highly susceptible to the cytopathic effect of a combined OVV and DOX treatment both *in vitro* and in syngeneic and xenograft tumor models.^9^ Consistent with the increased apoptotic effect mediated by the viral and DOX combination, our study with additional human OC cell lines and clinical specimens demonstrated significantly higher activation of CREB3L1 by the combined treatment compared to the single-treatment modalities. However, the effect of OVV on CREB3L1 expression and translocation was undetectable or small compared to the reported activation of CREB3L1 in responses to infection with RNA viruses (hepatitis C virus, West Nile virus, and Sendai virus) and murine γ-herpesvirus 68 DNA virus.^12^ This may be related to differences in the viral replication cycle and interaction with the host chaperone network required for entry, maturation, viral protein folding or assembly.^35^ For example, the WR strain of vaccinia used in our study displays no binding to ceramide, which *de novo* synthesis induces CREB3L1 activation,^36^ and also uses a unique viral assembly pathway.^37^ Unlike other enveloped viruses that acquire a single membrane by budding into a cellular organelle, vaccinia virus becomes enwrapped by a membrane cisterna that does not originate *de novo* but rather derives from the intermediate compartment between the ER and the Golgi stacks.^37^ It is also possible that deletions of *VGF* and *TK* genes, which are essential for replication of OVV in normal cells but not in cancer cells,^38^ influence the nuclear translocation of CREB3L1, which could also vary in different cancer cells.

Several lines of evidence indicate that type I IFN signaling is necessary for the efficacy of some chemotherapies^10^ and increased levels of type I IFN-regulated MxA protein closely relates to TIL infiltration and is an independent prognostic factor for DFS in triple-negative breast carcinoma.^39^ Our observation of increased responses to DOX treatment in the presence of IFN-β is consistent with previous studies that IFN-β production during virally induced ICD increases responses to DOX,^10^ supporting this mechanistic link following virus infection. The effect of IFN-β on CREB3L1 nuclear translocation when used with DOX may also have a clinical application by predicting more sustained responses to DOX in EOC patients because activation of the IFN-β/IFNAR/IRF7 signaling axis have been reported to instigate immunological dormancy in some chemotherapy-treated cancers,^40^ and the role of type I IFN in promoting response to chemotherapy in tumor cells has been recently reported.^10^^,^^41^

Our study showed that the expression levels of CREB3L1 in tumor cells cannot predict the response of EOC patients to DOX-based treatment based on a lack of correlation with OS, PFS, or TIL numbers. These findings differ from the previous studies suggesting that measurement of CREB3L1 expression could be a useful biomarker in identifying cancer cells sensitive to DOX.^13^^,^^14^^,^^18^ Among possible reasons for these discrepancies are differences in the chemotherapy regimens containing DOX in combination with other anti-cancer agents but not DOX alone.^14^ Consequently, in an event of the patient’s response to the treatment, it would be difficult to determine which anti-cancer drug mediated the effect. Furthermore, because DOX stimulates production of ceramide, which in turn triggers regulated intramembrane proteolysis (RIP) of CREB3L1, allowing the cleaved nuclear form of the protein to activate *p21* and other genes that inhibit cell proliferation,^12^^,^^14^ the overall sensitivity of cancer cells to DOX-mediated RIP might depend on generation of different signals in the ER to activate their unique set(s) of target genes.^42^ Consistent with this possibility are recent studies showing that the CREB3L1 involvement in tumor progression ranges from suppression^16^^,^^43^ to promotion of metastatic dissemination in tumors that have activated both protein kinase-like ER kinase (PERK) and have undergone an epithelial-to-mesenchymal transition (EMT) program.^44^ Thus, the role of CREB3L1 activation in tumor progression should be analyzed in a broader context of changes in cancer cells related to the expression of a mesenchymal phenotype of the tumor as well as signaling pathways including *CREB3L1* DNA methylation,^16^ alterations in histone modifications, regulation of RNA processing,^45^ and translational deregulation.^46^

It has been established that the interaction of DOX and oncolytic viruses mediate enhanced anticancer effects by both direct oncolysis and stimulation of innate immune responses through production of DAMPs and the presence of virus-derived PAMPs,^47^ leading to increased type 1 IFN production.^48^ In particular, vaccinia virus can be considered a suitable candidate for treatment of drug-resistant ovarian tumors owing to its ability to infect a broad range of cancer cells including cancer-initiating cells (CICs),^49^ a rapid replication cycle, production of extracellular enveloped virions that evade the immune response,^50^ and a capacity to spread to distant metastases following local delivery.^51^ We have previously demonstrated that OVV synergizes with DOX in the killing of paclitaxel- and carboplatin-resistant variants of OC with a phenotype characterized by augmented expression of the hyaluronan receptor CD44 and CXCR4 along with elevated Akt and ERK1/2 activation.^9^^,^^49^ The virus delivered to tumor-bearing mice prior to DOX treatment elicited a multifaceted response resulting in a synergistic killing of the resistant variants, decreases in intratumoral recruitment of immunosuppressive elements, and stimulation of antitumor immunity that led to inhibition of tumor growth.^9^ Taken together, the efficacious combination of OVV and DOX produces changes in CREB3L1 signaling pathways associated with the nuclear translocation of this factor in cancer cells and identify a mechanism of improving the effectiveness of DOX-based chemotherapy for EOC patients, while also providing the potent therapeutic benefits of viral oncotherapy.

## Materials and methods

### Cell lines and clinical specimens

OC patient tumors (FIGO stage IC–IV) were surgically removed at Roswell Park and received in the laboratory from tissue procurement under protocol I215512 which allows experimental use of patient tissue specimens for research. This study was approved by the Institutional Review Board (IRB) of Roswell Park Comprehensive Cancer Center (Buffalo, NY, USA) and was in compliance with federal and state requirements. Tumor samples were minced and mechanically disrupted with a Miltenyi gentleMACS tissue disruptor, followed by passage through a 300-μm strainer and centrifugation at 400 × *g* for 5 min. Cells were washed, counted, and used for ImageStream or RT-PCR analyses. An additional set of clinical specimens from 28 EOC patients with advanced stage of diseases (23 out of 28 patients were resistant to carboplatin) were analyzed for CREB3L1 expression by RNA-seq. Seventeen of these patients were on Doxil prior to entering clinical trials with anti-PD1 antibody. Human OC cell line SKOV3 was obtained from ATCC (Manassas, VA, USA), whereas cell lines A2780 and OVCA429 were obtained from Dr. Adam Karpf (Roswell Park Comprehensive Cancer Center, Buffalo, NY, USA). The cells were maintained in RPMI 1640 supplemented with 10% FBS as previously described.^52^ The authentication was done using short tandem repeat (STR) loci using AmpFlSTR profiler PCR amplification kit from Applied Biosystems (Foster City, CA, USA), which amplifies 15 STR loci and the amelogenin gene in one single multiplex PCR reaction and provides loci consistent with major world-wide STR databasing standards.

### Vaccinia virus

The vaccinia virus used is of the WR strain with disrupted *TK* and *VGF* genes for enhanced cancer cell specificity. The generation and characterization of this virus expressing the Fc portion of murine immunoglobulin (Ig)G2a has been described.^53^

### Viral- and DOX-induced cancer cell death

Adherent cultures from established cell lines were either infected with OVV at an MOI of 1 for 2 h, incubated with 1 μM DOX (Sigma, St. Louis, MO, USA) for 16 h, or treated with both consecutively. Cultures were detached with Accutase (Sigma, St. Louis, MO, USA) and stained with Live/Dead fixable violet (Thermo Fisher Scientific, Waltham, MA, USA) as per the manufacturer’s instruction. Cells were subsequently incubated with FITC-conjugated annexin V (BD Pharmingen, San Diego, CA, USA) according to the recommended procedure. Analysis was conducted on an LSRFortessa flow cytometer (BD Biosciences, San Jose, CA, USA) with FACSDiva acquisition software (BD Biosciences, San Jose, CA, USA). Data analysis was performed using WinList 9.0.1 (Verity Software House, Topsham, ME, USA).

### Cell quantification

The number of cells was determined by direct counting of adherent cells in untreated and treated cultures by trypan blue (Thermo Fisher Scientific, Waltham, MA, USA) exclusion and presented as the mean value from triplicate incubation.

### Western blot

Cell proteins were extracted by using cell lysis buffer (Cell Signaling Technology, Danvers, MA, USA) in the presence of protease inhibitor. The protein concentrations were determined by a Bradford assay. Proteins were separated by standard SDS-PAGE and transferred to nitrocellulose membranes (Bio-Rad, Hercules, CA, USA). Membranes were blocked using 5% milk (Cell Signaling Technology, Danvers, MA, USA), and expression of markers for apoptosis and necroptosis was analyzed by immunoblotting using the apoptosis/necroptosis antibody sampler kit (Cell Signaling Technology, Danvers, MA, USA) according to the manufacturer’s instruction. The primary antibodies included the following: rabbit monoclonal antibodies (mAbs) against RIP1 (D94C12), phosphorylated-RIP1 (Ser166) (D1L3S), caspase-3 (D3R6Y), cleaved caspase-3 (Asp175) (5A1E), and GAPDH (D16H11). Bands were visualized with horseradish peroxide (HRP)-linked secondary anti-rabbit IgG (Cell Signaling Technology, Danvers, MA, USA) followed by an enhanced chemiluminescence (ECL) western blotting detection system (Cytiva, Marlborough, MA, USA, USA).

### ImageStream analysis of CREB3L1 nuclear translocation

For ImageStream analysis of tumor specimens, leukocyte depletion was performed by magnetically labeling with CD45 microbeads (Miltenyi Biotec, Auburn, CA, USA), passing through an LS column (Miltenyi Biotec, Auburn, CA, USA) and collecting the negative fraction. The CD45-negative cancer cells from clinical specimens and adherent cultures from established cell lines (A2780, SKOV3, and OVCA429) were either infected with OVV at an MOI of 1 for 2 h, incubated with DOX (1 μM, for 1 h), or treated with both consecutively. In some experiments, recombinant IFN-β (100 pg/mL) was used instead of OVV. At the end of the 3-h incubation period, cultures were detached with Accutase and stained with Live/Dead fixable violet. Cells were fixed and permeabilized with eBioscience Foxp3/transcription factor fixation/permeabilization reagents (Thermo Fisher Scientific, Waltham, MA, USA) and incubated with FITC-conjugated rabbit anti-human CREB3L1 N-terminal region antibody (Aviva Systems Biology, San Diego, CA, USA, 1:100 dilution). Nuclei were stained with DRAQ5 fluorescent probe solution (Thermo Fisher Scientific, Waltham, MA, USA, 10 μM). Analysis was performed on an Amnis ImageStream-X MkII imaging flow cytometer (Luminex, Austin, TX, USA) using Inspire acquisition software (Luminex, Austin, TX, USA). Live/Dead fixable violet was excited at 405 nm and detected in channel 7, FITC at 488 nm in channel 2, DOX at 488 nm in channel 3, and DRAQ5 at 642 nm in channel 11. Only events that corresponded to single (based on brightfield area and aspect ratio), live (low Live/Dead stain), nucleated cells (DRAQ5 stain) were collected.

Following compensation of the spectral images based on a compensation matrix defined by single color controls, the degree of nuclear localization of CREB3L1 was measured using the “similarity” feature in the IDEAS software v6.2 (Luminex, Austin, TX, USA), as described.^31^^,^^54^ Briefly, for each cell a nuclear region of interest was defined (“masked”) using the default “morphology” mask of the IDEAS software applied to the DRAQ5 (channel 11) images. Next, a SS was applied to that nuclear mask for each cell for the corresponding FITC (CREB3L1, channel 2) and DRAQ5 (nucleus, channel 11) images. The SS is a log-transformed Pearson’s correlation coefficient between the pixel values of two image pairs and provides a quantitative measure of the degree of nuclear localization of the factor of interest (in this case CREB3L1). Cells with a low SS exhibit poor correlation between the images (corresponding with a predominant cytoplasmic distribution of the CREB3L1 factor), whereas cells with a high SS exhibit positive correlation between the images (corresponding with a predominant nuclear distribution of CREB3L1). The relative shift in this distribution between two populations (e.g., control versus treated cells) was calculated using the Rd value. Specifically, the mean SS of the test population (time points > 0) minus the mean SS of the control population (time = 0) then divided by the sum of the standard deviations of both populations.

### Quantitative real-time PCR

Total RNA was extracted from cells using the miRNeasy mini kit (QIAGEN, Germantown, MD, USA), and quantitative assessment of the purified total RNA was accomplished by using a Qubit broad range RNA kit (Thermo Fisher Scientific, Waltham, MA, USA). The RNA was further evaluated qualitatively using RNA Nanotape on the 4200 TapeStation (Agilent Technologies, Santa Clara, CA), where sizing of the RNA was determined, and a qualitative numerical score (RINe) was assigned. RNA was reverse transcribed to cDNA with a high-capacity cDNA reverse transcription kit (Applied Biosystems, Foster City, CA, USA). CREB3L1 expression was measured by quantitative real-time PCR performed using Power SYBR Green master mix (Life Technologies, Grand Island, NY, USA) according to the manufacturer’s protocol. qRT-PCR was performed using utilizing a forward primer (5′-CCTTGTGCTTTGTTCTGGTG-3′) and reverse primer (5′-GTAGAATAGGAGGCTTCGGG-3′), which amplifies a 103-bp fragment at the junction of exons 8 and 9. Relative expression was calculated using GAPDH as a reference gene rather than β-actin gene because of more consistent cycle threshold (CT) values for GAPDH compared to those for β-actin, which were highly variable across the analyzed specimens. Samples were analyzed in technical triplicate per reaction on the Applied Biosystems QuantStudio 6 flex real-time PCR system and analyzed with Applied Biosystems QuantStudio real-time PCR software v1.2. Relative change in expression was calculated using ΔΔCT method.^55^ The average of ΔCT values for all samples was used to calculate ΔΔCT.

### RNA-seq data

OC samples were mapped to hg38 human reference genome and GENCODE (v25) annotation database using TopHat (v2.0.13) with a maximum of one mismatch per read. The aligned reads were further checked with RSeQC (v2.6.3) in order to identify potential library preparation-related problems. Gene level read counts were estimated with featureCounts from Subread (v1.6.0) using the -fracOverlap 1 option. Raw read counts were normalized at the gene level using DESeq2’s (v1.26) median of ratios procedure accounting for library size; in particular, CREB3L1 expression values were derived from this estimation.

### Survival analysis using TCGA-OV dataset

TCGA gene expression dataset was downloaded from cBioPortal using ovarian serous cystadenocarcinoma (TCGA PanCancer Atlas, 300 samples with RNA-seq). TCGA OC patients were ranked based on the expression value of CREB3L1 and grouped in the top versus bottom tertile (group of high expression versus group of low expression of CREB3L1). Survival analyses, including OS, DFS, and PFS, were performed using the survival package in R.

### Statistical analysis

Statistical analyses were performed using GraphPad Prism 6 (GraphPad, La Jolla, CA, USA). Unless otherwise noted, the data were summarized as mean ± SD, combined with a two-tailed Student’s t test to compare two groups or one-way analysis of variance (ANOVA) for data analysis of more than two groups followed by a Bonferroni multiple comparison test. Spearman correlation was used to test between two continuous variables. Two-group survival statistical testing was done fitting a Cox proportional hazards model and summarized using Kaplan-Meier curves. The threshold for statistical significance was set to p < 0.05. All statistical tests are two-tailed unless otherwise specified. A p value less than 0.05 was considered significant.

## Supplemental information

Document S1. Supplemental materials and methods and Figures S1–S4

Document S2. Article plus supplemental information

## References

[1] Stewart C., Ralyea C., Lockwood S. (2019). Ovarian cancer: An integrated review. Semin. Oncol. Nurs..

[2] Fujiwara K., Hasegawa K., Nagao S. (2019). Landscape of systemic therapy for ovarian cancer in 2019: Primary therapy. Cancer.

[3] Karam A., Ledermann J.A., Kim J.W., Sehouli J., Lu K., Gourley C., Katsumata N., Burger R.A., Nam B.H., Bacon M., participants of the 5th Ovarian Cancer Consensus Conference (2017). Fifth Ovarian Cancer Consensus Conference of the Gynecologic Cancer InterGroup: First-line interventions. Ann. Oncol..

[4] Chatterjee K., Zhang J., Honbo N., Karliner J.S. (2010). Doxorubicin cardiomyopathy. Cardiology.

[5] Foley O.W., Rauh-Hain J.A., del Carmen M.G. (2013). Recurrent epithelial ovarian cancer: An update on treatment. Oncology (Williston Park).

[6] Hanahan D., Weinberg R.A. (2011). Hallmarks of cancer: The next generation. Cell.

[7] Lawler S.E., Speranza M.C., Cho C.F., Chiocca E.A. (2017). Oncolytic viruses in cancer treatment: A review. JAMA Oncol..

[8] Pisano C., Cecere S.C., Di Napoli M., Cavaliere C., Tambaro R., Facchini G., Scaffa C., Losito S., Pizzolorusso A., Pignata S. (2013). Clinical trials with pegylated liposomal doxorubicin in the treatment of ovarian cancer. J. Drug Deliv..

[9] Komorowski M.P., McGray A.R., Kolakowska A., Eng K., Gil M., Opyrchal M., Litwinska B., Nemeth M.J., Odunsi K.O., Kozbor D. (2016). Reprogramming antitumor immunity against chemoresistant ovarian cancer by a CXCR4 antagonist-armed viral oncotherapy. Mol. Ther. Oncolytics.

[10] Sistigu A., Yamazaki T., Vacchelli E., Chaba K., Enot D.P., Adam J., Vitale I., Goubar A., Baracco E.E., Remédios C. (2014). Cancer cell-autonomous contribution of type I interferon signaling to the efficacy of chemotherapy. Nat. Med..

[11] Honma Y., Kanazawa K., Mori T., Tanno Y., Tojo M., Kiyosawa H., Takeda J., Nikaido T., Tsukamoto T., Yokoya S., Wanaka A. (1999). Identification of a novel gene, OASIS, which encodes for a putative CREB/ATF family transcription factor in the long-term cultured astrocytes and gliotic tissue. Brain Res. Mol. Brain Res..

[12] Denard B., Seemann J., Chen Q., Gay A., Huang H., Chen Y., Ye J. (2011). The membrane-bound transcription factor CREB3L1 is activated in response to virus infection to inhibit proliferation of virus-infected cells. Cell Host Microbe.

[13] Denard B., Pavia-Jimenez A., Chen W., Williams N.S., Naina H., Collins R., Brugarolas J., Ye J. (2015). Identification of CREB3L1 as a biomarker predicting doxorubicin treatment outcome. PLoS ONE.

[14] Denard B., Lee C., Ye J. (2012). Doxorubicin blocks proliferation of cancer cells through proteolytic activation of CREB3L1. eLife.

[15] Denard B., Jiang S., Peng Y., Ye J. (2018). CREB3L1 as a potential biomarker predicting response of triple negative breast cancer to doxorubicin-based chemotherapy. BMC Cancer.

[16] Ward A.K., Mellor P., Smith S.E., Kendall S., Just N.A., Vizeacoumar F.S., Sarker S., Phillips Z., Alvi R., Saxena A. (2016). Epigenetic silencing of CREB3L1 by DNA methylation is associated with high-grade metastatic breast cancers with poor prognosis and is prevalent in triple negative breast cancers. Breast Cancer Res..

[17] Rose M., Schubert C., Dierichs L., Gaisa N.T., Heer M., Heidenreich A., Knüchel R., Dahl E. (2014). *OASIS/CREB3L1* is epigenetically silenced in human bladder cancer facilitating tumor cell spreading and migration in vitro. Epigenetics.

[18] Xiao W., Liang Y., Que Y., Li J., Peng R., Xu B., Wen X., Zhao J., Guan Y., Zhang X. (2019). Comparison of the MAID (AI) and CAV/IE regimens with the predictive value of cyclic AMP-responsive element-binding protein 3 like protein 1 (CREB3L1) in palliative chemotherapy for advanced soft-tissue sarcoma patients. J. Cancer.

[19] Guo Z.S., Liu Z., Kowalsky S., Feist M., Kalinski P., Lu B., Storkus W.J., Bartlett D.L. (2017). Oncolytic immunotherapy: Conceptual evolution, current strategies, and future perspectives. Front. Immunol..

[20] van Vloten J.P., Workenhe S.T., Wootton S.K., Mossman K.L., Bridle B.W. (2018). Critical interactions between immunogenic cancer cell death, oncolytic viruses, and the immune system define the rational design of combination immunotherapies. J. Immunol..

[21] Woo S.R., Corrales L., Gajewski T.F. (2015). The STING pathway and the T cell-inflamed tumor microenvironment. Trends Immunol..

[22] Odunsi K., Sabbatini P. (2008). Harnessing the immune system for ovarian cancer therapy. Am. J. Reprod. Immunol..

[23] Crowley L.C., Marfell B.J., Scott A.P., Waterhouse N.J. (2016). Quantitation of apoptosis and necrosis by annexin V binding, propidium iodide uptake, and flow cytometry. Cold Spring Harb. Protoc..

[24] Whilding L.M., Archibald K.M., Kulbe H., Balkwill F.R., Öberg D., McNeish I.A. (2013). Vaccinia virus induces programmed necrosis in ovarian cancer cells. Mol. Ther..

[25] Guo Z.S., Lu B., Guo Z., Giehl E., Feist M., Dai E., Liu W., Storkus W.J., He Y., Liu Z., Bartlett D.L. (2019). Vaccinia virus-mediated cancer immunotherapy: Cancer vaccines and oncolytics. J. Immunother. Cancer.

[26] Dhuriya Y.K., Sharma D. (2018). Necroptosis: A regulated inflammatory mode of cell death. J. Neuroinflammation.

[27] Crowley L.C., Waterhouse N.J. (2016). Detecting cleaved caspase-3 in apoptotic cells by flow cytometry. Cold Spring Harb. Protoc..

[28] Greiner S., Humrich J.Y., Thuman P., Sauter B., Schuler G., Jenne L. (2006). The highly attenuated vaccinia virus strain modified virus Ankara induces apoptosis in melanoma cells and allows bystander dendritic cells to generate a potent anti-tumoral immunity. Clin. Exp. Immunol..

[29] Liskova J., Knitlova J., Honner R., Melkova Z. (2011). Apoptosis and necrosis in vaccinia virus-infected HeLa G and BSC-40 cells. Virus Res..

[30] O’Leary M.P., Warner S.G., Kim S.I., Chaurasiya S., Lu J., Choi A.H., Park A.K., Woo Y., Fong Y., Chen N.G. (2018). A novel oncolytic chimeric orthopoxvirus encoding luciferase enables real-time view of colorectal cancer cell infection. Mol. Ther. Oncolytics.

[31] Maguire O., Collins C., O’Loughlin K., Miecznikowski J., Minderman H. (2011). Quantifying nuclear p65 as a parameter for NF-κB activation: Correlation between ImageStream cytometry, microscopy, and western blot. Cytometry A.

[32] Guo Z.S., Naik A., O’Malley M.E., Popovic P., Demarco R., Hu Y., Yin X., Yang S., Zeh H.J., Moss B. (2005). The enhanced tumor selectivity of an oncolytic vaccinia lacking the host range and antiapoptosis genes SPI-1 and SPI-2. Cancer Res..

[33] Galluzzi L., Kepp O., Morselli E., Vitale I., Senovilla L., Pinti M., Zitvogel L., Kroemer G. (2010). Viral strategies for the evasion of immunogenic cell death. J. Intern. Med..

[34] Kettle S., Blake N.W., Law K.M., Smith G.L. (1995). Vaccinia virus serpins B13R (SPI-2) and B22R (SPI-1) encode *M*_r_ 38.5 and 40K, intracellular polypeptides that do not affect virus virulence in a murine intranasal model. Virology.

[35] Feng J., Gong D., Fu X., Wu T.T., Wang J., Chang J., Zhou J., Lu G., Wang Y., Sun R. (2015). M1 of murine gamma-herpesvirus 68 induces endoplasmic reticulum chaperone production. Sci. Rep..

[36] Perino J., Foo C.H., Spehner D., Cohen G.H., Eisenberg R.J., Crance J.M., Favier A.L. (2011). Role of sulfatide in vaccinia virus infection. Biol. Cell.

[37] Sodeik B., Doms R.W., Ericsson M., Hiller G., Machamer C.E., van ’t Hof W., van Meer G., Moss B., Griffiths G. (1993). Assembly of vaccinia virus: Role of the intermediate compartment between the endoplasmic reticulum and the Golgi stacks. J. Cell Biol..

[38] McCart J.A., Ward J.M., Lee J., Hu Y., Alexander H.R., Libutti S.K., Moss B., Bartlett D.L. (2001). Systemic cancer therapy with a tumor-selective vaccinia virus mutant lacking thymidine kinase and vaccinia growth factor genes. Cancer Res..

[39] Kim Y.A., Lee H.J., Heo S.H., Park H.S., Park S.Y., Bang W., Song I.H., Park I.A., Gong G. (2016). MxA expression is associated with tumor-infiltrating lymphocytes and is a prognostic factor in triple-negative breast cancer. Breast Cancer Res. Treat..

[40] Lan Q., Peyvandi S., Duffey N., Huang Y.T., Barras D., Held W., Richard F., Delorenzi M., Sotiriou C., Desmedt C. (2019). Type I interferon/IRF7 axis instigates chemotherapy-induced immunological dormancy in breast cancer. Oncogene.

[41] Legrier M.E., Bièche I., Gaston J., Beurdeley A., Yvonnet V., Déas O., Thuleau A., Château-Joubert S., Servely J.L., Vacher S. (2016). Activation of IFN/STAT1 signalling predicts response to chemotherapy in oestrogen receptor-negative breast cancer. Br. J. Cancer.

[42] Ye J. (2020). Transcription factors activated through RIP (regulated intramembrane proteolysis) and RAT (regulated alternative translocation). J. Biol. Chem..

[43] Mellor P., Deibert L., Calvert B., Bonham K., Carlsen S.A., Anderson D.H. (2013). CREB3L1 is a metastasis suppressor that represses expression of genes regulating metastasis, invasion, and angiogenesis. Mol. Cell. Biol..

[44] Feng Y.X., Jin D.X., Sokol E.S., Reinhardt F., Miller D.H., Gupta P.B. (2017). Cancer-specific PERK signaling drives invasion and metastasis through CREB3L1. Nat. Commun..

[45] Doma M.K., Parker R. (2007). RNA quality control in eukaryotes. Cell.

[46] Kondo S., Hino S.I., Saito A., Kanemoto S., Kawasaki N., Asada R., Izumi S., Iwamoto H., Oki M., Miyagi H. (2012). Activation of OASIS family, ER stress transducers, is dependent on its stabilization. Cell Death Differ..

[47] Lichty B.D., Breitbach C.J., Stojdl D.F., Bell J.C. (2014). Going viral with cancer immunotherapy. Nat. Rev. Cancer.

[48] Unterholzner L., Keating S.E., Baran M., Horan K.A., Jensen S.B., Sharma S., Sirois C.M., Jin T., Latz E., Xiao T.S. (2010). IFI16 is an innate immune sensor for intracellular DNA. Nat. Immunol..

[49] Gil M., Komorowski M.P., Seshadri M., Rokita H., McGray A.J., Opyrchal M., Odunsi K.O., Kozbor D. (2014). CXCL12/CXCR4 blockade by oncolytic virotherapy inhibits ovarian cancer growth by decreasing immunosuppression and targeting cancer-initiating cells. J. Immunol..

[50] Vanderplasschen A., Mathew E., Hollinshead M., Sim R.B., Smith G.L. (1998). Extracellular enveloped vaccinia virus is resistant to complement because of incorporation of host complement control proteins into its envelope. Proc. Natl. Acad. Sci. USA.

[51] Park B.H., Hwang T., Liu T.C., Sze D.Y., Kim J.S., Kwon H.C., Oh S.Y., Han S.Y., Yoon J.H., Hong S.H. (2008). Use of a targeted oncolytic poxvirus, JX-594, in patients with refractory primary or metastatic liver cancer: A phase I trial. Lancet Oncol..

[52] Woloszynska-Read A., James S.R., Link P.A., Yu J., Odunsi K., Karpf A.R. (2007). DNA methylation-dependent regulation of BORIS/CTCFL expression in ovarian cancer. Cancer Immun..

[53] Gil M., Seshadri M., Komorowski M.P., Abrams S.I., Kozbor D. (2013). Targeting CXCL12/CXCR4 signaling with oncolytic virotherapy disrupts tumor vasculature and inhibits breast cancer metastases. Proc. Natl. Acad. Sci. USA.

[54] Maguire O., O’Loughlin K., Minderman H. (2015). Simultaneous assessment of NF-κB/p65 phosphorylation and nuclear localization using imaging flow cytometry. J. Immunol. Methods.

[55] Livak K.J., Schmittgen T.D. (2001). Analysis of relative gene expression data using real-time quantitative PCR and the 2^−ΔΔC(T)^ method. Methods.

