# Supplementary material for: Induction of cell death in ovarian cancer cells by doxorubicin and oncolytic vaccinia virus is associated with CREB3L1 activation
Source: Mol Ther Oncolytics. 2021 Apr 29;23:38–50. doi: 10.1016/j.omto.2021.04.014 (PMC8479291; doi:10.1016/j.omto.2021.04.014)
Supplement: Document S1. Supplemental materials and methods and Figures S1–S4 [file mmc1.pdf]

OMTO, Volume *23*

Supplemental information

Induction of cell death in ovarian cancer cells by doxorubicin and oncolytic vaccinia virus is associated with CREB3L1 activation

Anna Mistarz, Matthew Graczyk, Marta Winkler, Prashant K. Singh, Eduardo Cortes, Anthony Miliotto, Song Liu, Mark Long, Li Yan, Aimee Stablewski, Kieran O'Loughlin, Hans Minderman, Kunle Odunsi, Hanna Rokita, A.J. Robert McGray, Emese Zsiros, and Danuta Kozbor

Supplemental Methods

Western blot analysis

Cell homogenates were separated into nuclear and cytoplasmic fractions using Nuclear (Nucleic Acid-Free) Extraction Kit (Abcam, Cambridge, MA). Equal amount of protein, determined by Bradford assay was separated by standard SDS-PAGE and transferred to nitrocellulose membranes (Bio-Rad, Hercules, CA). Membranes were blocked using 5% milk (Cell Signaling Technology, Danvers, MA) and incubated at 4°C overnight with primary antibodies against CREB3L1 (1:1000, Aviva Systems Biology, San Diego, CA) and the nuclear protein PCNA (1:1000, Abcam, Cambridge, MA). Bands were visualized with horseradish peroxide (HRP)-linked secondary antibodies (Cell Signaling Technology, Danvers, MA) followed by ECL Western Blotting detection system (Cytiva, Marlborough, MA).

ELISA

Adherent cultures from established cell lines were infected with OVV at multiplicity of infection (MOI) of 1. Cell culture supernatants were collected after 48 h and evaluated for presence of human IFN-β by ELISA (R&D Systems, Minneapolis, MN) according to manufacturer's protocol.

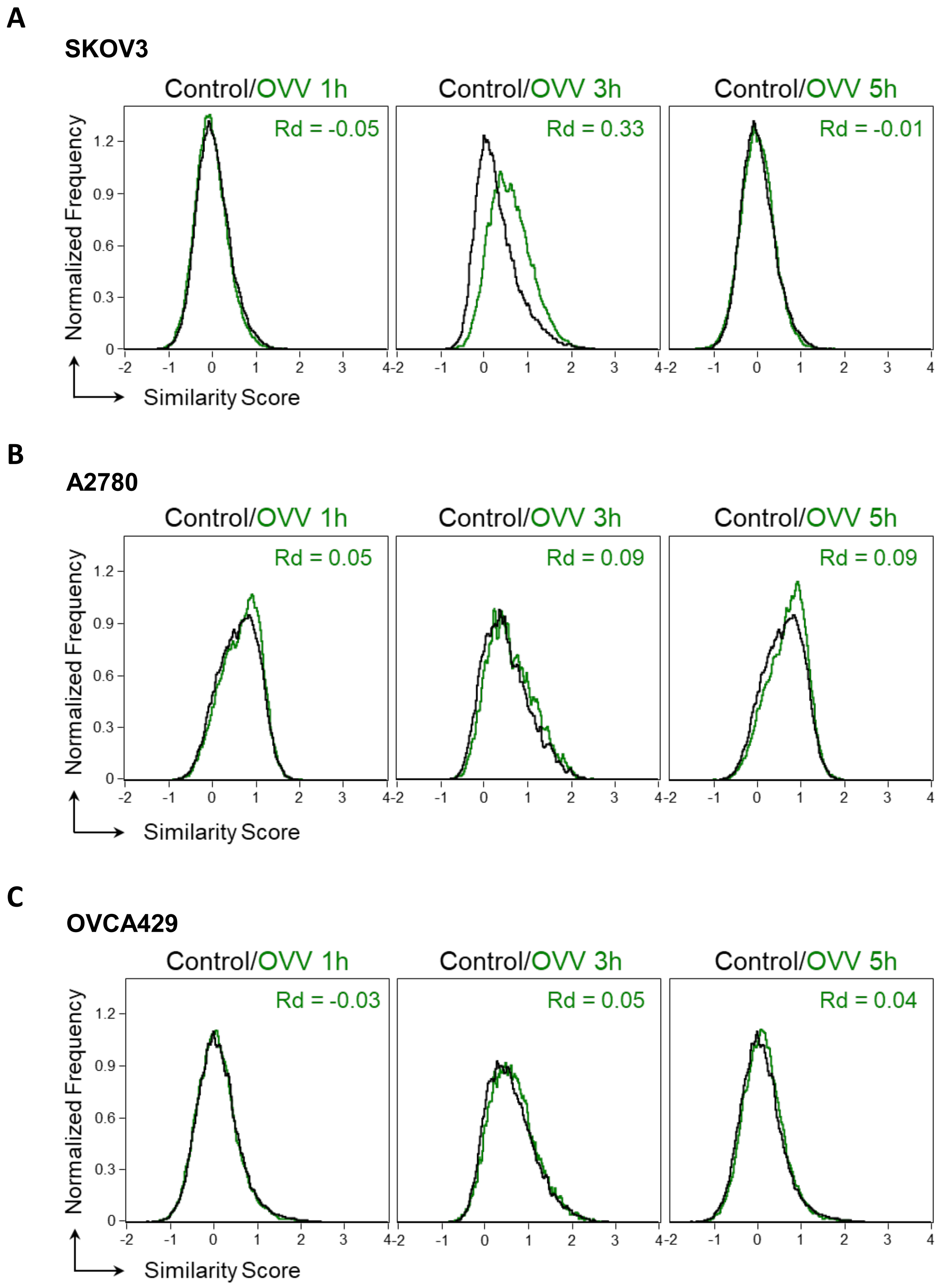

Figure S1. Time-dependent analysis of nuclear translocation of CREB3L1. SKOV3 (**A**), A2780 (**B**) and OVCA429 (**C**) cells were infected with OVV (MOI = 1) and analyzed for nuclear translocation of CREB3L1 by ImageStream. Distributions of cells with varying SSs are graphed with the Rd values shown for each population. Results are representative of three independent experiments.

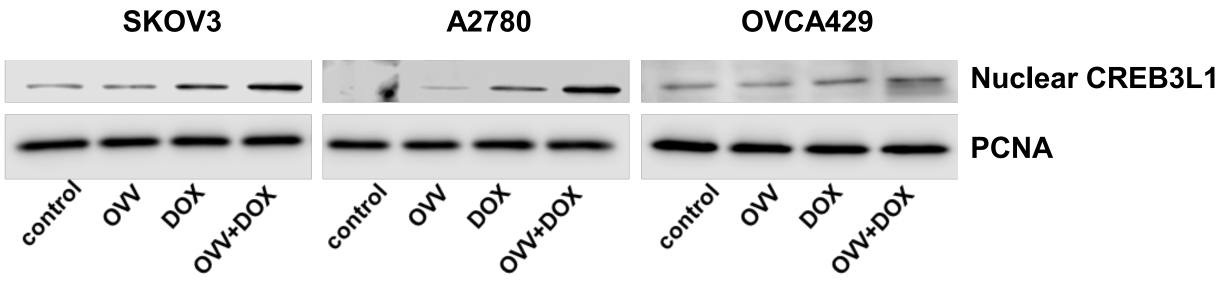

Figure S2. Western blot analysis of nuclear translocation of CREB3L1 in SKOV3, A2780 and OVCA429 cells after treatment with OVV or DOX alone or in combination. Cells were treated as described in the Materials and Methods section. Nuclear fraction of the cells was then analyzed by immunoblotting with antibody reacting against CREB3L1 and PCNA used as a loading control for nuclear extract and density of the CREB3L1 bands. Results of two independent experiments are shown.

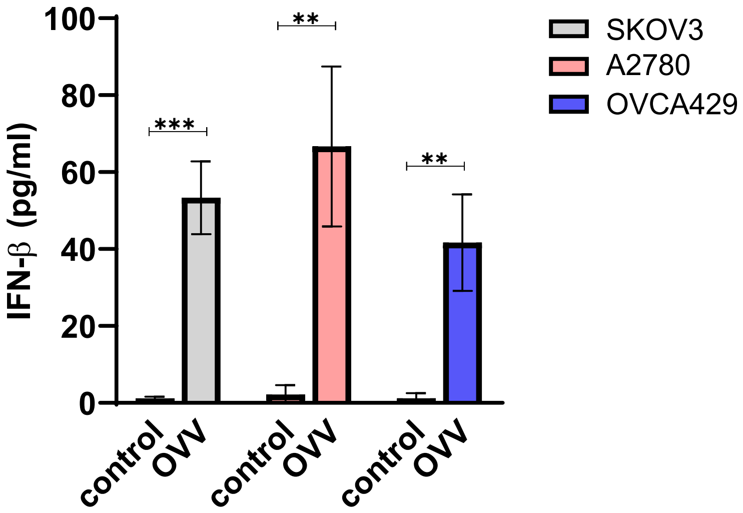

Figure S3. IFN-β production in SKOV3, A2780 and OVC429 cell cultures after infection with OVV. Cells were infected for 2 h with OVV (MOI = 1) and cultured for 48 h. The amounts of human IFN-β in cell culture supernatans were determined by ELISA. Results are presented as mean ± of two independent experiments.

A

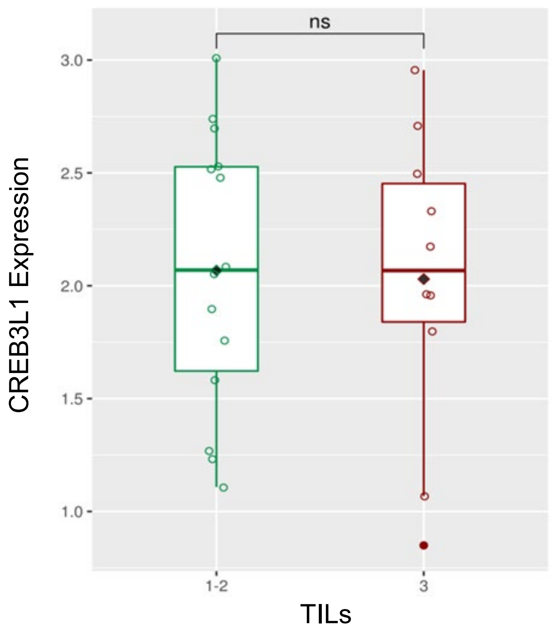

B

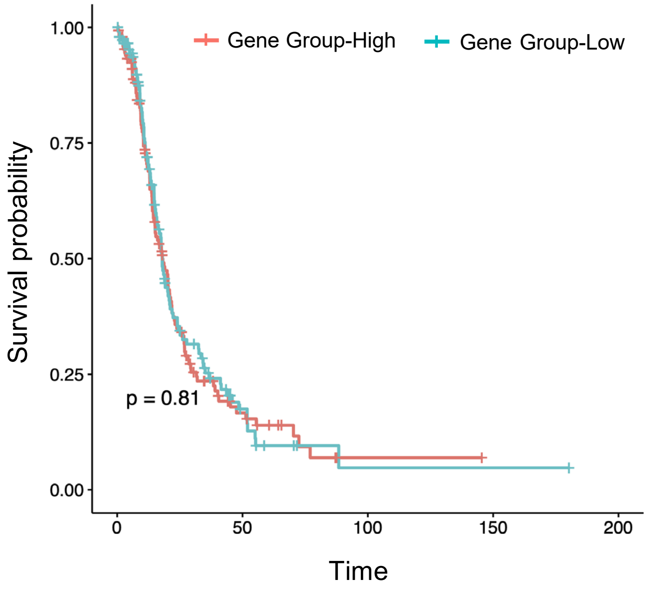

Figure S4. RNAseq analyses of CREB3L1 expression in ovarian tumor data. (A) Comparison of CREB3L1 expression levels between TILs groups of a cohort of patients of 28 EOC patients with advanced stage of diseases with seventeen of these patients treated with Doxil prior to entering clinical trials with anti-PD1 antibody. (**B)** Kaplan-Meier curves describing PFS among EOC patients from TCGA data based. CREB3L1 overexpression (red line) was not associated with higher PFS (0.81).
